# Association between Meal Frequency and Type 2 Diabetes Mellitus in Rural Adults: A Large-Scale Cross-Sectional Study

**DOI:** 10.3390/nu15061348

**Published:** 2023-03-10

**Authors:** Bota Baheti, Xiaotian Liu, Mu Wang, Caiyun Zhang, Xiaokang Dong, Ning Kang, Linlin Li, Xing Li, Songcheng Yu, Jian Hou, Zhenxing Mao, Chongjian Wang

**Affiliations:** 1 Department of Epidemiology and Biostatistics, College of Public Health, Zhengzhou University, Zhengzhou 450001, China; 2 Clinical Mass Spectrometry Laboratory, Clinical Research Institute, Affiliated Nanhua Hospital, Hengyang Medical College, University of South China, Hengyang 421200, China; 3 Department of Nutrition and Food Hygiene, College of Public Health, Zhengzhou University, Zhengzhou 450001, China

**Keywords:** meal frequency, dinner frequency, type 2 diabetes, rural population, stratified analysis

## Abstract

Diet frequency may potentially influence metabolic health. However, general population-based evidence on the association between meal frequency and type 2 diabetes mellitus (T2DM) remains limited and inconclusive. Thus, this study aimed to investigate the association between meal frequency and T2DM in resource limited area. A total of 29,405 qualified participants were enrolled from the Henan rural cohort study. Data on meal frequency were collected through a validated face-to-face questionnaire survey. Logistic regression models were utilized to explore the association between meal frequency and T2DM. Compared with 21 times per week meal frequency group, the adjusted odds ratios (*ORs*) and 95% confidence intervals (*95%CIs*) were 0.75 (0.58, 0.95) and 0.70 (0.54, 0.90) for 16–20 times/week group and 14–15 times/week group, respectively. For the analysis of the three meals, significant associations were only found between dinner frequency and T2DM. Compared with seven times per week dinner group, the *ORs* (*95%CIs*) were 0.66 (0.42, 0.99) and 0.51 (0.29, 0.82) for the group with three to six times/week and zero to two times/week. Reduced meal frequency, especially dinner frequency, was associated with lower prevalence of T2DM, which suggests that an appropriate reduction in meal frequency per week may have a role in decreasing the risk of T2DM.

## 1. Introduction

Type 2 diabetes mellitus (T2DM), one of the metabolic diseases featured by high blood glucose levels, has emerged as a prominent public health problem around the world. According to the International Diabetes Federation (IDF), the worldwide prevalence of diabetes has reached 10.5% in 2021, meaning that 537 million adults living with diabetes, and the fraction may climb to 783 million by 2045 [1,2]. Diabetes imposed tremendous socioeconomic pressure on individuals and healthcare systems around the world. The global health expenditure caused by diabetes was estimated to be $966 billion United States dollars (USD) in 2021, a threefold increase from 15 years ago. Importantly, more than three-quarters of people with diabetes live in low and middle-income countries [3]. China, a representative of developing countries, still has large populations living in rural areas. Age-standardized prevalence of T2DM in rural areas of China has been estimated at 6.98% and it has still been escalating. In contrast to urban populations, rural populations have a higher prevalence of T2DM, but lower rates of treatment and control owing to the lack of medical service resources and health care system coverage [4,5]. Therefore, identifying the potentially harmful behaviors and adopting convenient and effective interventions for preventing the incidence and development of diabetes in rural populations may significantly alleviate the national and family burden, and this also has considerable value for reducing the overall prevalence of diabetes.

Apart from the traditional factors, diet behavior as a modifiable factor is of increasing attention due to its potential impact on human health, especially on diseases relating to metabolism [6]. Nevertheless, research on this topic has produced inconclusive results. A prospective study conducted on Chinese community residents showed that increasing diet frequency may reduce the risk of T2DM [7]. Another study of American health professional subjects indicated that lower eating frequency was associated with a higher risk of T2DM [8]. Besides, previous studies have suggested that smaller and more frequent meals in an isocaloric condition were beneficial in improving metabolic health among obese patients with prediabetes [9]. However, it has been argued that this benefit was limited, and it was worth noting that increasing diet frequency in free-living populations might induce excessive energy intake, while frequent high-calorie diets, especially high-energy dinners, might increase the risk of metabolic syndrome [10,11]. Gradually, concentration has shifted to reducing the frequency of meals and then extending the duration of fasting between two meals, which may promote metabolic health [12,13,14]. For example, a study on American professional women revealed that the risk of T2DM was higher among participants who consumed breakfast irregularly but with higher total diet frequency, compared to those who consumed breakfast regularly but with lower total diet frequency, when classifying participants according to their breakfast consumption and eating frequency [15]. In addition, another study of postmenopausal women from American clinical centers found that higher diet frequency may elevate the risk of T2DM [16]. Furthermore, there were also some epidemiological studies indicating that appropriate caloric restriction (CR) might be helpful for improving glycemic control [17] and cardiometabolic status [18], and this might prevent the majority of chronic diseases. Moreover, several animal and clinical studies have shown that intermittent fasting (IF), a special form of reducing diet frequency, could also confer benefits for many health conditions, including improvements in blood lipids, blood pressure, glucose homeostasis, insulin resistance, metabolic disorder, and even might help delay aging and extend the life span [19,20,21,22,23,24,25,26]. However, most of the above studies were experimental studies or mainly focused on developed countries and urban settings, the general population-based evidence of the association between meal frequency and T2DM in resource-limited areas is still limited. In view of the cultural diversity of food between Western countries and China, and the differences in economic development, education level, medical resources, lifestyle behaviors, food choices, and dietary patterns between urban and rural populations in China, it has great practical importance to explore the health effect of modifiable dietary factors in rural population from the resource-limited area in the context of the increasing burden of T2DM.

Therefore, this study aimed to explore the association between meal frequency and T2DM in rural populations of the resource-limited area, and then it investigated whether these associations were different among those in different breakfast, lunch, dinner frequencies and other subgroups.

## 2. Methods

### 2.1. Study Population

Participants in the study were recruited from the Henan Rural Cohort study (Registration number: ChiCTR-OOC-15006699). Detailed information on the methodology of the Henan Rural Cohort has been previously reported [27]. Briefly, 39,259 permanent populations between the ages of 18–79 years were recruited from five rural areas of Henan (Tongxu, Yuzhou, Suiping, Xinxiang, and Yima) via multistage stratified cluster sampling from July 2015 to September 2017. This cross-sectional study included the following exclusion criteria: (1) miss information on meal frequency (*n* = 9293); (2) lack of information on T2DM (*n* = 57); (3) type 1 diabetes mellitus (*n* = 4); (4) implausible meal frequency (*n* = 190). (5) participants with cancer (*n* = 292) and kidney failure (*n* = 18). Finally, a total of 29,405 subjects were enrolled in this current study, and details can be found in Figure S1. All of the participants supplied written informed consent, and the researchers followed the guidelines issued by the Declaration of Helsinki.

### 2.2. Assessment of Meal Frequency

A validated standardized questionnaire was used to obtain information on meal frequency by well-trained investigators through a face-to face interview (Cronbach’s alpha coefficient, α = 0.729, intraclass correlation coefficients, ICC = 0.841) [27]. The definition of meal frequency in this questionnaire was based on customary or regular main meals that contained staple foods, and snacks that did not contain staple foods, such as beverages and chips, were not considered as a meal and were not collected and included in the analysis. Participants were required to answer the questions below: “In the past week, how many times did you normally eat breakfast, lunch, or dinner?” and then asked, “how many times did you normally eat breakfast, lunch, or dinner away from home each week?” Then, the total weekly meal frequency for each participant was calculated by adding the weekly frequency of breakfast, lunch, and dinner. The distribution of total weekly meal frequency in the analyzed sample ranged from 14–21 times/week. So, the total weekly meal frequency was divided into two groups of 21 times/week (normal meal frequency group) and 14–20 times/week (reduced meal frequency group). Afterwards, in order to explore whether this relationship would be enhanced with decreasing meal frequency, weekly meal frequency was further divided into three groups of 14–15 times/week, 16–20 times/week, and 21 times/week, and the trend test was performed with the highest group as the reference. In parallel, the frequency of breakfast, lunch, and dinner was categorized into seven times/week (normal meal frequency group) and zero to six times/week (reduced meal frequency group), then the breakfast, lunch, and dinner frequency were also classified into zero to two times/week, three to six times/week, and seven times/week in further analysis.

### 2.3. Definition of T2DM

After overnight fasting for >8 h, blood samples were sampled, and fasting glucose (FBG) was measured with an automated biochemical analyzer (Cobas c501, Roche, Switzerland). Participants who met the following criteria were considered to have T2DM: FBG ≥ 7.0 mmol/L, or previously diagnosed with T2DM by a physician, and taking insulin/oral hypoglycemic agents during the last two weeks.

### 2.4. Covariates Estimate

Dietary intake information on individuals was obtained by well trained staff through a 13-item validated food frequency questionnaire (FFQ) that has been shown to have good reproducibility and validity [28]. The FFQ covered the frequency and amount of 13 types of food consumed in the past year, namely, staple foods, livestock, poultry, fish, eggs, milk, fruits, vegetables, legumes, nuts, preserved products, grains, and animal oils. Then, the total energy intake of each participant was computed based on the China Food Composition Table 2004. Abundant vegetable and fruit intake were identified as consumption of more than 500 g of vegetable and fruit each day. Consumption of fat over 75 g each day was considered a high-fat diet. Consumption of salt over 2 g each day was classified as a high-salt diet. Eating away from home was considered as consuming meals prepared outside the home, and then weekly eating away from home frequency was computed by adding the frequency of breakfast, lunch, and dinner eating out each week.

Non-dietary covariates were also collected by validated standardized questionnaires with good validity and reliability [27], including demographic variables (age and gender), social–economic status (educational level, average monthly income, and marital status), lifestyle covariates (smoking and drinking status, physical activity), and family history of T2DM. Briefly, this current study age was grouped into <60 years and ≥60 years. There were two levels of marital status: married/cohabiting and widowed/divorced/separated/single. Educational level was classified into those three grades: elementary school or below, junior high school, and senior high school or above. Average monthly income was classified into <500 RMB, 500-RMB, and 1000-RMB. According to drinking and smoking status, participants were divided into non-drinker, drinker, non-smoker, and smoker, respectively. According to the International Physical Activity Questionnaire (IPAQ), physical activity was categorized into three levels: low, moderate, and high. The basal metabolic rate and body weight of subjects was measured by OMRONV. BODY HBF-371 instrument followed the operational instructions. BMI was counted as weight (kg) divided by the square of height. All of the above measurements were carried out by well trained staff following standardized procedures.

### 2.5. Statistical Analysis

For descriptive analysis of participants, continuous variables and categorical variables were expressed as mean ± standard deviation (SD) and quantity (proportion), respectively. Student’s *t*-test and chi-squared test were used to compare differences in continuous and categorical variables between T2DM and non-T2DM groups, respectively.

Logistic regression analysis was utilized to estimate the association of meal frequency and T2DM risk by the *ORs* and *95%CIs*. The three models were fitted as follows: Model 1 was unadjusted; Model 2 was only adjusted for age and gender; Model 3 was adjusted for age, gender, marital status, average monthly income, education level, smoking status, drinking status, physical activity, vegetables and fruits intake, high-fat diet, high-salt diet, BMI, total energy intake, basal metabolic rate, family history of T2DM, and weekly frequency of eating away from home. The estimated effect of each time reduction of meal frequency was explored with the highest meal frequency as the reference. Then, trend analysis was performed to estimate the association between each level reduction of meal frequency and T2DM by treating the categorical variables as continuous variables in the logistic regression. In addition, stratified analyses were performed in different gender, ages, BMI, smoking and drinking status, high-fat diet, high-salt diet, and vegetable and fruit intake. The highest categories—21 times/week (total meal frequency) and seven times/week (breakfast, lunch, dinner)—were considered as the reference groups in all analyses.

All analyses were accomplished with Statistical Package for the Social Sciences version 21.0 (IBM-SPSS Inc., Armonk, NY, USA) and R software version 4.0.3. Two-tailed *p* values < 0.05 were considered statistically significant.

## 3. Results

### 3.1. Basic Characteristics of Participants

Table 1 depicted the basic characteristic of the subjects. A total of 29,405 participants, aged 55.48 ± 12.32 years old, were recruited in this study, including 12,022 (40.88%) men and 17,383 (59.12%) women. Of these participants, 2585 had T2DM with a crude prevalence rate of 8.79%. Subjects identified with T2DM are more likely to have lower family income, education levels, ratios of smoking, physical activity, vegetable or fruit intake, and high-fat diet, as well as higher levels of age, BMI, basal metabolic rate, and family history of diabetes and so on. What is more, the mean frequency of weekly total meal, breakfast, and dinner were higher for participants with T2DM than for non-T2DM (all *p* < 0.05).

### 3.2. Association between the Weekly Total Meal Frequency and T2DM

The association between weekly total meal frequency and T2DM was presented in Table 2 and Supplemental Table S1. The findings showed a positive association between weekly total meal frequency and T2DM. Firstly, when the participants were classified into reduced meal frequency group (14–20 times/week) and regular meal frequency group (21 times/week), the adjusted *OR* (*95%CIs*) was 0.73 (0.60, 0.87) for 14–20 times/week, compared with 21 times/week group. When the 29,405 participants were further divided into three groups, the *ORs* (*95%CIs*) of 16–20 times/week and 14–15 times/week were 0.75 (0.58, 0.95) and 0.70 (0.54, 0.90) compared with the reference group (21 times/week) after controlling multiple variables in model 3. Besides, the adjusted *OR* (*95%CIs*) for each time reduction of meal frequency was 0.95 (0.92, 0.98). Additionally, the adjusted *OR* (*95%CIs*) for each level reduction in total meal frequency was 0.82 (0.73, 0.92), and the *p* value of the trend test was 0.001.

### 3.3. Association between the Weekly Frequency of Breakfast, Lunch, and Dinner and T2DM

The impacts of the breakfast, lunch, and dinner frequency on T2DM were shown in Table 2 and Supplemental Table S1. However, with the exception of dinner, no significant association between the frequency of breakfast or lunch and T2DM was found. When the participants were classified into reduced dinner frequency group and regular dinner frequency group, the adjusted *OR* (*95%CIs*) was 0.59 (0.42, 0.81) for zero to six times/week compared with those who consumed dinner seven times/week in model 3 (Supplemental Table S1). When further analysis was performed by dividing into three groups, the adjusted *ORs* (*95%CIs*) were 0.66 (0.42, 0.99) and 0.51 (0.29, 0.82) for participants who consumed dinner three to six times/week and zero to two times/week compared with seven times/week, respectively. In addition, the *ORs* and *95%CIs* for each time reduction of dinner frequency was 0.89 (0.83, 0.96), Furthermore, the *ORs* (*95%CIs*) for each level reduction in the frequency of dinner was 0.70 (0.56, 0.87), and the *p* value for the trend test was 0.002 on the fully adjusted model.

### 3.4. Stratified Analysis

**Figure 1:**
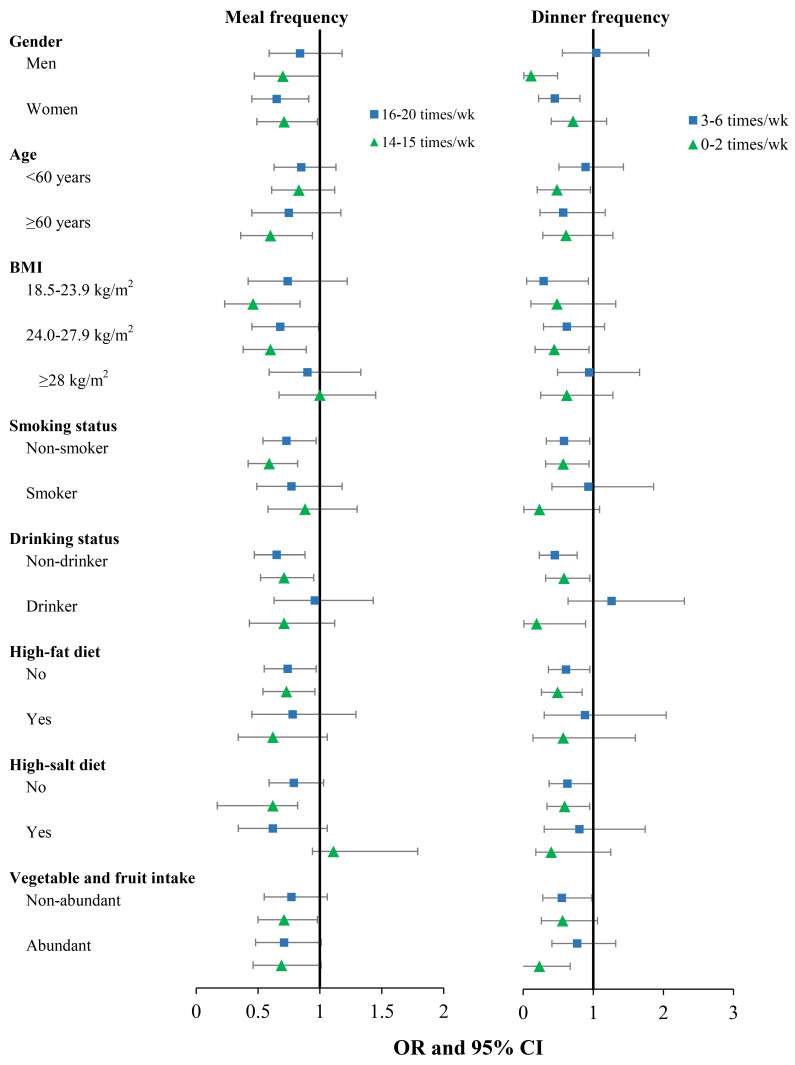
Stratified analysis of the association between weekly meal frequency, dinner frequency and T2DM. Notes: 21 times per week and seven times per week as references, respectively.

Figure 1 was drawn based on Supplemental Tables S2 and S3. In stratified analysis, significant associations between weekly total meal frequency and T2DM were found among participants who were women, age ≥ 60 years old, BMI < 28 kg/m^2^, do not smoke or drink, having a non-high fat diet, having a non-high salt diet, and having non-abundant vegetable and fruit intake (Figure 1). To be specific, in model 3, the *ORs* and *95%CIs* of 16–20 times/week and 14–15 times/week were 0.65 (0.45, 0.91) and 0.71 (0.49, 0.98) among women. Besides, the *ORs* and *95%CIs* of 14–15 times/week was 0.60 (0.36, 0.94) among participants who were ≥60 years. Additionally, the *ORs* and *95%CIs* was 0.46 (0.23, 0.84) for normal body mass index subjects with meal frequency of 14–15 times/week, the *ORs* and *95%CIs* were 0.68 (0.45, 0.99) and 0.60 (0.38, 0.89) for overweight participants with meal frequency of 16–20 times/week and 14–15 times/week, respectively (P_interaction_ = 0.027). Furthermore, the *ORs* and *95%CIs* were 0.73 (0.54, 0.97) and 0.59 (0.42, 0.82) for non-smokers with meal frequency of 16–20 times/week and 14–15 times/week (P_interaction_ = 0.016). Moreover, the *ORs* and *95%CIs* were 0.65 (0.47, 0.88) and 0.71 (0.52, 0.95) for non-drinkers with meal frequency of 16–20 times/week and 14–15 times/week. The *ORs* and *95%CIs* were 0.74 (0.55, 0.97) and 0.73 (0.54, 0.96) for non-high fat diet participants with meal frequency of 16–20 times/week and 14–15 times/week. Furthermore, the *ORs* and *95%CIs* was 0.62 (0.45, 0.82) for non-high salt diet participants with meal frequency of 14–15 times/week and 0.71 (0.50, 0.98) for non-abundant vegetable and fruit intake participants with meal frequency of 14–15 times/week. Briefly, in the stratified analysis of weekly dinner frequency (Figure 1), significant associations were found in both men and women, age < 60 years old, BMI < 28 kg/m^2^, participants who do not smoke or drink, non-high fat diet or non-high salt diet consumers, abundant or non-abundant fruit and vegetable consumers.

## 4. Discussion

This large-scale cross-sectional study explored the association between meal frequency and the risk of T2DM in rural adults from the resource-limited area. The results of this study indicated that a reduced meal frequency was associated with a lower prevalence of T2DM. In addition, as for the analysis of three meals, a reduced dinner frequency was significantly associated with a lower prevalence of T2DM. Furthermore, in stratified analyses, significant associations between meal frequency and T2DM were found among participants with relatively healthy lifestyles. The findings of this current study provide meaningful information for the primary prevention of T2DM through dietary frequency changes in resource-limited populations.

Studies on diet frequency and T2DM risk have yielded inconsistent results. A follow-up study of American health professional men showed that lower eating frequency was associated with a higher risk of T2DM [8]. Besides, another study conducted on Chinese community residents showed that eating four meals a day was related to a lower risk of T2DM compared to eating three meals a day, while eating two meals a day showed no relationship with T2DM [7]. In addition, there was a randomized trial focusing on prediabetes patients, suggesting that increasing the frequency of eating under isocaloric conditions can provide a variety of metabolic benefits, including improved glucose metabolism [9]. However, it seems very difficult to maintain a constant total energy intake in free-living populations who self-reported diets, and even a higher frequency of eating could lead to a higher calorie intake. This was because frequent eating might increase the stimulation of food and result in more energy intake, thus making it difficult to control energy balance [29,30,31,32]. Furthermore, there was also a study pointing out that changing eating frequency had virtually no effect on glucose regulation parameters, but consumption of most calories in the evening of the day might be harmful to glycemic control [33]. All these discrepant findings are possibly due to different study populations, lifestyles behaviors, dietary habits, sample sizes, definitions of diet frequency, methods for assessing meal frequency, and different adjusted covariates. In this current study, we found a positive association between meal frequency and T2DM, and it was in alignment with some previous studies. For instance, a prospective study of older women reported that, compared with three times per day, relative risks and 95% confidence intervals were 1.13 (1.00, 1.27) for participants who ate four to five times per day. Additionally, participants who ate breakfast irregularly, but with higher total eating frequency, were at a greater risk of T2DM (RR: 1.47, 95% CI: 1.23, 1.75) compared with those who ate breakfast daily but with lower eating frequency (one to three times per day) [15]. Furthermore, another cohort study focused on postmenopausal women found that hazard rates and 95% confidence intervals were 1.38 (1.03, 1.84) for subjects who consumed meals four times per day compared with one to three times per day [16]. Moreover, a study based on an Iranian population showed that those who have six meals per day are at a higher risk of diabetes than those who eat three meals per day (OR: 2.503, 95% CI: 1.651, 3.793) [34]. Additionally, there were several studies that also revealed that plenty of health parameters could be improved by reducing energy intake, including glycemic control, β cell function, insulin resistance, lipid profiles, oxidative stress, and inflammation [19,21,24,35].

In this study, no significant association between breakfast or lunch frequency and T2DM was found. Similar to our results, a prospective study based on the frequency of breakfast and the risk of T2DM among community-dwelling older adults also did not observe significant associations [36]. Unfortunately, as for lunch frequency, the number of participants in the group of zero to two times/week and three to six times/week is small, so results on lunch frequency might be unreliable. Due to the insufficient sample size, we did not further explore the association between lunch frequency and T2DM. However, we found that reduced dinner frequency per week to prolong the duration of the overnight fast was significantly associated with a lower prevalence of T2DM in this study. Although few studies have examined the relationship between dinner frequency and T2DM, there was still some evidence that could support the viewpoints of this study. For instance, a randomized crossover study comparing the effect of two meals (only breakfast and lunch) versus six meals a day demonstrated that a low-energy diet pattern of two meals per day reduced fasting plasma glucose, glucagon, C-peptide, and hepatic fat content, and it improved insulin sensitivity [37]. Besides, some recent studies focusing on early time-restricted eating patterns (eTRE) illustrated that consuming food at earlier times of the day (dinner was not eaten) and extending the length of the night fast could produce metabolic health improvements, including weight loss, improved insulin sensitivity, fasting insulin, and reduced serum glucose excursions after glucose loading in healthy individuals, as well as subjects with prediabetes [38,39]. Moreover, previous studies also have shown that high energy intake at dinner was associated with an increased incidence of diabetes, as well as increased mortality from diabetes and cardiovascular disease and all-cause mortality in people with diabetes [40,41]. Taken all together, reducing energy intake at dinner may be beneficial for metabolic health to some extent.

In stratified analysis, we found that this association was significant among subjects who had relatively healthy lifestyles. Several studies have shown that smoking and drinking were risk factors for diabetes [42,43]. Furthermore, high-fat diets and high-salt diets have been shown to affect the metabolism of glucose and lipids, impair the function of major metabolic organs, and subsequently increase the risk of diabetes [44,45]. Taken together, these poor lifestyles might weaken the beneficial effects of reduced diet frequency. Moreover, this study found no significant association between meal frequency and T2DM among participants with BMI ≥ 28 kg/m^2^. It might be that these populations had multiple adverse physical conditions, such as chronic inflammation, dyslipidemia, impaired postprandial metabolism, or insulin resistance, which could potentially diminish the benefits of reduced meal frequency [46,47].

Overall, there are several potential mechanisms that might explain this association. First, metabolic conversion from glucose to fatty acid-derived ketones not only provided ketones required by cells during fasting, but also elicited a highly coordinated systemic and cellular response that could enhance resistance to disease [48,49]. Second, intermittent fasting activated adaptive cellular stress response signaling pathways, thereby enhancing mitochondrial health, DNA repair, and autophagy [50,51,52,53]. Third, intermittent fasting promoted the production of brain-derived neurotrophic factors, which increased neuronal resistance to dysfunction and degeneration and regulated the glucose metabolism disorder caused by its deficiency [48,54]. Fourth, caloric restriction and reduced eating frequency could increase antioxidant activity and reduce oxidative stress, thus preventing many diseases caused by oxidative stress, including diabetes [22,38,55,56].

This present study has a couple of limitations needing attention. Firstly, this was a cross-sectional study, so, we could not ascertain the causal relationship between meal frequency and T2DM. Therefore, long-term longitudinal studies are needed to validate this association. Secondly, since meal frequency was obtained by asking each participant about diets in the past week through recall, there might be recall bias due to participants’ unclear recall of past diets. However, FFQ and the standardized questionnaire used in this current study were attested to previously have good reproducibility and validity in our previous research [28]. Thirdly, some details about eating habits were not fully obtained, for example, snacking behavior, the time of a meal, and the quality of foods consumed with each meal, all of which might influence the association in this current study [8,57,58]. However, in contrast to Western populations, Chinese middle-aged and elderly consumers usually eat three regular main meals per day and might rarely eat snacks. Therefore, three main meals may account for the majority of daily energy intake [59]. In addition, according to our survey, snacking behavior was relatively not popular in rural areas, and eating away from home was more prevalent than snacking behavior, so we collected and adjusted for the covariates of eating out frequency, high-fat diet, high-salt diet, vegetable and fruit intake status, basal metabolic rate, and total energy intake to minimize this confounding effect as far as possible. Finally, even though this study adjusted for many potential confounders, the effect of unmeasured residual confounders could not be completely ruled out. Therefore, further research on the effects of the above factors on T2DM is required in the future.

## 5. Conclusions

In summary, meal frequency might be a potentially modifiable factor for T2DM, and a positive association was observed between meal frequency and T2DM. The current study suggested that appropriately reducing the meal frequency, especially the dinner frequency, may be beneficial in the prevention of T2DM. In the future, large and long-term prospective studies are needed to verify this association.

**Research in context:**

**What is already known about this subject?**

- Dietary behaviors as a modifiable factor might have potential effects on metabolic health.
- Previous studies have shown that reducing diet frequency (including intermittent fasting, calorie restriction) and then extending the fasting period might have beneficial effects on health conditions.

**What is the key question?**

- Evidence on the health effects of intermittent fasting or calorie restriction comes mainly from a few experimental studies with a small sample and short duration.
- General population-based evidence of reducing diet frequency was still limited and inconclusive, especially lacking evidence from low and middle-income areas.

**What are the new findings?**

- Intermittent reduction in meal frequency was associated with a lower prevalence of T2DM.
- Moderating the frequency of dinner might reduce the risk of T2DM.
- Those associations were significant among participants with relatively healthy lifestyles.

## Figures and Tables

**Table 1:** Basic characteristics of participants according to T2DM (*n* = 29,405).

Variables	Total (*n* = 29,405)	T2DM (*n* = 2585)	Non-T2DM (*n* = 26,820)	*p*-Value
Age (years) (mean ± SD)	55.48 ± 12.32	60.37 ± 9.42	55.01 ± 12.47	<0.001 ^a^
Gender, *n* (%)				0.060 ^b^
Men	12,022 (40.88)	1012 (39.15)	11,010 (41.05)	
Women	17,383 (59.12)	1573 (60.85)	15,810 (58.95)	
Marital status, *n* (%)				0.132 ^b^
Married/cohabitating	26,524 (90.2)	2310 (89.36)	24,214 (90.28)	
Unmarried/divorced/widowed	2881 (9.80)	275 (10.64)	2606 (9.72)	
Average monthly income, *n* (%)				<0.001 ^b^
<500 RMB	10,607 (36.07)	1030 (39.85)	9577 (35.71)	
500-RMB	9278 (31.55)	796 (30.79)	8482 (31.63)	
1000-RMB	9520 (32.38)	759 (29.36)	8761 (32.67)	
Education level, *n* (%)				<0.001 ^b^
Elementary school or below	13,055 (44.40)	1408 (54.47)	11,647 (43.43)	
Junior high school	11,431 (38.87)	858 (33.19)	10,573 (39.42)	
High school or above	4919 (16.73)	319 (12.34)	4600 (17.15)	
Smoking status, *n* (%)				0.002 ^b^
Non-smokers	21,150 (71.93)	1928 (74.58)	19,222 (71.67)	
Smokers	8255 (28.07)	657 (25.42)	7598 (28.33)	
Drinking status, *n* (%)				0.084 ^b^
Non-drinkers	22,819 (77.60)	2041 (78.96)	20,778 (77.47)	
Drinkers	6586 (22.40)	544 (21.04)	6024 (22.53)	
Physical activity, *n* (%)				<0.001 ^b^
Low	9326 (31.72)	971 (37.56)	8355 (31.15)	
Moderate	10,821 (36.80)	865 (33.46)	9956 (37.12)	
High	9258 (31.48)	749 (28.97)	8509 (31.73)	
High-salt diet, *n* (%)	4751 (16.16)	435 (16.85)	4316 (16.12)	0.335 ^b^
Abundant vegetable and fruit intake, *n* (%)	14,097 (74.94)	1117 (43.21)	12,980 (48.40)	<0.001 ^b^
High-fat diet, *n* (%)	5343 (18.17)	388 (15.01)	4955 (18.48)	<0.001 ^b^
BMI (kg/m^2^), (mean ± SD)	24.73 ± 3.56	26.19 ± 3.67	24.59 ± 3.52	<0.001 ^a^
Total energy intake (kcal/d)	2433.02 ± 676.15	2377.60 ± 682.41	2438.36 ± 675.32	<0.001 ^a^
Basal metabolic rate (KJ/m^2^/h) (mean ± SD)	1378.39 ± 214.35	1411.18 ± 223.73	1375.25 ± 213.17	<0.001 ^a^
Family history of T2DM, *n* (%)	1111 (3.78)	253 (9.79)	858 (3.20)	<0.001 ^b^
FBG (mmol/L, mean ± SD)	5.51 ± 1.47	9.00 ± 2.82	5.17 ± 0.58	<0.001 ^a^
Total meal frequency (times/week)	20.53 ± 1.59	20.71 ± 1.27	20.51 ± 1.62	<0.001 ^a^
Breakfast frequency (times/week)	6.66 ± 1.38	6.80 ± 1.08	6.64 ± 1.40	<0.001 ^a^
Lunch frequency (times/week)	6.98 ± 0.28	6.98 ± 0.36	6.98 ± 0.27	0.411 ^a^
Dinner frequency (times/week)	6.89 ± 0.76	6.94 ± 0.58	6.89 ± 0.77	<0.001 ^a^

Continuous variables are presented as mean ± SD; categorical variables are shown as quantity (percentages). ^a^ *p*-values were from Student’s *t*-tests for continuous data. ^b^ *p*-values were from the chi-squared test for categorical data. BMI: body mass index; SD standard deviation; T2DM: type 2 diabetes mellitus. FBG: fasting blood glucose.

**Table 2:** Multivariate-adjusted ORs and *95%CIs* for T2DM according to weekly meal frequency.

Variables	*OR* (*95% CI*)				* p _trend_
	Model 1	Model 2	Model 3	* Per Level Risk	
**Total meal frequency**				0.82 (0.73–0.92)	0.001
21 times/week (*n* = 26,621)	1.00 (Ref.)	1.00 (Ref.)	1.00 (Ref.)		
16–20 times/week (*n* = 1440)	0.55 (0.43–0.69)	0.83 (0.64–1.04)	0.75 (0.58–0.95)		
14–15 times/week (*n* = 1344)	0.57 (0.45–0.72)	0.81 (0.63–1.02)	0.70 (0.54–0.90)		
Each time reduction	0.91 (0.88–0.94)	0.97 (0.94–1.00)	0.95 (0.92–0.98)		
**Breakfast frequency**				0.89 (0.79–1.02)	0.090
7 times/week (*n* = 27,359)	1.00 (Ref.)	1.00 (Ref.)	1.00 (Ref.)		
3–6 times/week (*n* = 861)	0.53 (0.38–0.71)	0.86 (0.62–1.16)	0.81 (0.58–1.11)		
0–2 times/week (*n* = 1185)	0.55 (0.42–0.71)	0.86 (0.65–1.11)	0.83 (0.63–1.08)		
Each time reduction	0.90 (0.87–0.94)	0.97 (0.94–1.01)	0.97 (0.93–1.01)		
**Lunch frequency**				1.08 (0.72–1.62)	0.703
7 times/week (*n* = 29,195)	1.00 (Ref.)	1.00 (Ref.)	1.00 (Ref.)		
3–6 times/week (*n* = 172)	0.51 (0.23–0.96)	0.69 (0.31–1.31)	0.64 (0.28–1.24)		
0–2 times/week (*n* = 38)	2.34 (0.95–5.01)	2.34 (0.93–5.12)	2.18 (0.80–5.05)		
Each time reduction	1.06 (0.93–1.20)	1.08 (0.96–1.23)	1.06 (0.93–1.22)		
**Dinner frequency**				0.70 (0.56–0.87)	0.002
7 times/week (*n* = 28,621)	1.00 (Ref.)	1.00 (Ref.)	1.00 (Ref.)		
3–6 times/week (*n* = 463)	0.56 (0.36–0.83)	0.76 (0.49–1.13)	0.66 (0.42–0.99)		
0–2 times/week (*n* = 321)	0.57 (0.34–0.91)	0.62 (0.37–0.99)	0.51 (0.29–0.82)		
Each time reduction	0.89 (0.83–0.96)	0.92 (0.86–0.99)	0.89 (0.83–0.96)		

*OR*: odds ratio; *CI:* confidence interval. * Full-adjusted model for age, gender, marital status, average monthly income, education level, smoking status, drinking status, physical activity, vegetable and fruit intake, high-fat diet, high-salt diet, BMI, total energy intake, basal metabolic rate, family history of T2DM, and weekly frequency of eating-out (total meals, breakfast, lunch, and dinner). Trends of odds ratios were performed using the categories (≥21 times, 16–20 times, 14–15 times or seven times, three to six times and zero to two times) of weekly meal frequency as a continuous variable in the logistic regression model, respectively. Model 1 was unadjusted; Model 2 was adjusted for age and gender; Model 3 was adjusted for age and gender, marital status, average monthly income, education level, smoking status, drinking status, physical activity, vegetable and fruit intake, high-fat diet, high-salt diet, BMI, energy intake, basal metabolic rate, family history of T2DM, and weekly frequency of eating-out (total meals, breakfast, lunch, and dinner).

## Data Availability

The data analyzed during current study are available from the corresponding author upon reasonable request. Contact Chongjian Wang (tjwcj2008@zzu.edu.cn) for additional information regarding data access.

## Supplementary Materials

The following supporting information can be downloaded at: https://www.mdpi.com/article/10.3390/nu15061348/s1, Figure S1: The flow chart for selecting study population; Table S1: Multivariate-adjusted ORs and 95% CI for T2DM according to meal frequency; Table S2: Stratified analysis of the association between total meal frequency and T2DM; Table S3: Stratified analysis of the association between dinner frequency and T2DM.

## Abbreviations

T2DM:	type 2 diabetes mellitus
FBG:	fasting blood glucose
IDF:	International Diabetes Federation
FFQ:	food frequency questionnaire
IPAQ:	International Physical Activity Questionnaire
BMI:	body mass index
SD:	standard deviation
ORs:	odds ratios
*95%CIs*:	95% confidence intervals
RR:	risk ratio
IF:	intermittent fasting
TRE:	time-restricted eating patterns
