# Supplementary material for: Association between Meal Frequency and Type 2 Diabetes Mellitus in Rural Adults: A Large-Scale Cross-Sectional Study
Source: Nutrients. 2023 Mar 10;15(6):1348. doi: 10.3390/nu15061348 (PMC10056094; doi:10.3390/nu15061348)
Supplement: Supplementary file 1 [file nutrients-15-01348-s001.zip › nutrients-2180761-supplementary.pdf]

ELECTRONIC SUPPLEMENTARY DATA

Title

Association between Meal Frequency and Type 2 Diabetes Mellitus in Rural Adults: A Large-Scale Cross-Sectional Study

Authors:

Bota Baheti [1,†], Xiaotian Liu [1,†], Mu Wang [2], Caiyun Zhang [1], Xiaokang Dong [1], Ning Kang [1], Linlin Li [1], Xing Li [3], Songcheng Yu [3], Jian Hou [1], Zhenxing Mao [1] and Chongjian Wang [1,*]

Authors affiliations:

[1] Department of Epidemiology and Biostatistics, College of Public Health, Zhengzhou University, Zhengzhou 450001, China; botabht@163.com (B.B.); xtliu2008@126.com (X.L.); z201528383@163.com (C.Z.); dong4568529173@163.com (X.D.); kangning97@163.com (N.K.); lilinlin563@163.com (L.L.); 13667176505@163.com (J.H.); maozhr@gmail.com (Z.M.)
[2] Clinical Mass Spectrometry Laboratory, Clinical Research Institute, Affiliated Nanhua Hospital, Hengyang Medical College, University of South China, Hengyang 421200, China; wangmu2009@163.com
[3] Department of Nutrition and Food Hygiene, College of Public Health, Zhengzhou University, Zhengzhou, China; lixing530@zzu.edu.cn (X.L.); scyu@zzu.edu.cn (S.Y.)
* Correspondence: tjwcj2008@zzu.edu.cn; Tel.: +86-371-6778-1452; Fax: +86-371-6778-1919
† These authors contributed equally to this work.

Conflict of interest: All authors declare that they have no conflict of interest.

Ethics approval:

Ethics approval was obtained from the “Zhengzhou University Life Science Ethics Committee”, and written informed consent was obtained for all participants. Ethic approval code: [2015] MEC (S128).

Consent to participant

Written consent was acquired from each participant prior to this study.

Supplemental Table S1. Multivariate-adjusted *ORs* and *95% CI* for T2DM according to meal frequency

OR: odds ratio; *CI:* confidence interval

**Variables**	***OR (95% CI)***		
	**Model 1**	**Model 2**	**Model 3**
**Total meal frequency**			
21 times/week (*n*=26,621)	1.00 (Ref.)	1.00 (Ref.)	1.00 (Ref.)
14-20 times/week (*n*=2784)	0.56 (0.47-0.66)	0.82 (0.68-0.97)	0.73 (0.60-0.87)
**Breakfast frequency**			
7 times/week (*n*=27,359)	1.00 (Ref.)	1.00 (Ref.)	1.00 (Ref.)
0-6 times/week (*n*=2046)	0.54 (0.44-0.66)	0.86 (0.69-1.05)	0.82 (0.66-1.01)
**Lunch frequency**			
7 times/week (*n*=29,195)	1.00 (Ref.)	1.00 (Ref.)	1.00 (Ref.)
0-6 times/week (*n*=210)	0.80 (0.45-1.30)	1.02 (0.57-1.67)	0.92 (0.50-1.55)
**Dinner frequency**			
7 times/week (*n*=28,621)	1.00 (Ref.)	1.00 (Ref.)	1.00 (Ref.)
0-6 times/week (*n*=784)	0.57 (0.41-0.77)	0.70 (0.50-0.95)	0.59 (0.42-0.81)

Model 1 was unadjusted;

Model 2 was adjusted for age and gender;

Model 3 was adjusted for age and gender, marital status, average monthly income, education level, smoking status, drinking status, physical activity, vegetable and fruit intake, high-fat diet, high-salt diet, BMI, energy intake, basal metabolic rate, family history of T2DM, weekly frequency of eating-out (total meal, breakfast, lunch, dinner).

Supplemental Table S2. Stratified analysis of the association between total meal frequency and T2DM

**Variables**	**Weekly total meal frequency**			***P*** *interaction*
	**21 times/week (*n*=26,621)**	**16-20 times/week (*n*=1440)**	**14-15 times/week (*n*=1344)**	
**Gender**				0.108
Men	1.00 (Ref.)	0.84 (0.59-1.18)	0.70 (0.47-1.00)	
Women	1.00 (Ref.)	0.65 (0.45-0.91)	0.71 (0.49-0.98)	
**Age**				0.136
< 60 years	1.00 (Ref.)	0.85 (0.63-1.13)	0.83 (0.61-1.12)	
≥ 60 years	1.00 (Ref.)	0.75 (0.45-1.17)	0.60 (0.36-0.94)	
**BMI**				0.027
18.5-23.9 kg/m^2	1.00 (Ref.)	0.74 (0.42-1.22)	0.46 (0.23-0.84)	
24.0-27.9 kg/m^2	1.00 (Ref.)	0.68 (0.45-0.99)	0.60 (0.38-0.89)	
≥ 28 kg/m^2	1.00 (Ref.)	0.90 (0.59-1.33)	1.00 (0.67-1.45)	
**Smoking status**				0.016
Non-smoker	1.00 (Ref.)	0.73 (0.54-0.97)	0.59 (0.42-0.82)	
Smoker	1.00 (Ref.)	0.77 (0.49-1.18)	0.88 (0.58-1.30)	
**Drinking status**				0.367
Non-drinker	1.00 (Ref.)	0.65 (0.47-0.88)	0.71 (0.52-0.95)	
Drinker	1.00 (Ref.)	0.96 (0.63-1.43)	0.71 (0.43-1.12)	
**High-fat diet**				0.556
No	1.00 (Ref.)	0.74 (0.55-0.97)	0.73 (0.54-0.96)	
Yes	1.00 (Ref.)	0.78 (0.45-1.29)	0.62 (0.34-1.06)	
**High-salt diet**				0.072
No	1.00 (Ref.)	0.79 (0.59-1.03)	0.62 (0.45-0.82)	
Yes	1.00 (Ref.)	0.62 (0.34-1.06)	1.11 (0.66-1.79)	
**Vegetable and fruit intake**				0.904
Non-abundant	1.00 (Ref.)	0.77 (0.55-1.06)	0.71 (0.50-0.98)	
Abundant	1.00 (Ref.)	0.71 (0.48-1.01)	0.69 (0.46-1.01)	

Full-adjusted model for age, gender, marital status, average monthly income, education level, smoking status, drinking status, physical activity, vegetable and fruit intake, high-fat diet, high-salt diet, BMI, energy intake, basal metabolic rate, family history of T2DM, weekly frequency of eating out

Supplemental Table S3. Stratified analysis of the association between dinner frequency and T2DM

Variables	Dinner frequency			$P_{interaction}$
	**7 times/week (*n*=28,621)**	**3-6 times/week (*n*=463)**	**0-2 times/week (*n*=321)**	
**Gender**				0.852
Men	1.00 (Ref.)	1.04 (0.56-1.79)	0.11 (0.01-0.49)	
Women	1.00 (Ref.)	0.45 (0.22-0.81)	0.71 (0.40-1.19)	
**Age**				0.234
< 60 years	1.00 (Ref.)	0.89 (0.51-1.43)	0.48 (0.20-0.96)	
≥ 60 years	1.00 (Ref.)	0.57 (0.24-1.17)	0.61 (0.28-1.18)	
**BMI**				0.117
18.5-23.9 kg/m^2	1.00 (Ref.)	0.29 (0.05-0.93)	0.48 (0.11-1.32)	
24.0-27.9 kg/m^2	1.00 (Ref.)	0.62 (0.29-1.16)	0.44 (0.17-0.94)	
≥ 28 kg/m^2	1.00 (Ref.)	0.94 (0.49-1.66)	0.62 (0.25-1.28)	
**Smoking status**				0.783
Non-smoker	1.00 (Ref.)	0.58 (0.33-0.95)	0.57 (0.32-0.94)	
Smoker	1.00 (Ref.)	0.93 (0.41-1.86)	0.23 (0.01-1.09)	
**Drinking status**				0.421
Non-drinker	1.00 (Ref.)	0.45 (0.23-0.77)	0.58 (0.32-0.95)	
Drinker	1.00 (Ref.)	1.26 (0.64-2.30)	0.19 (0.01-0.89)	
**High-fat diet**				0.569
No	1.00 (Ref.)	0.61 (0.36-0.95)	0.49 (0.26-0.84)	
Yes	1.00 (Ref.)	0.88 (0.30-2.04)	0.57 (0.14-1.60)	
**High-salt diet**				0.660
No	1.00 (Ref.)	0.63 (0.37-0.99)	0.56 (0.31-0.92)	
Yes	1.00 (Ref.)	0.80 (0.30-1.74)	0.23 (0.01-1.08)	
**Vegetable and fruit intake**				0.953
Non-abundant	1.00 (Ref.)	0.55 (0.28-0.98)	0.59 (0.29-1.09)	
Abundant	1.00 (Ref.)	0.77 (0.41-1.32)	0.40 (0.15-0.84)	

Full-adjusted model for age, gender, marital status, average monthly income, education level, smoking status, drinking status, physical activity, vegetable and fruit intake, high-fat diet, high-salt diet, BMI, energy intake, basal metabolic rate, family history of T2DM, weekly frequency of eating-out dinner

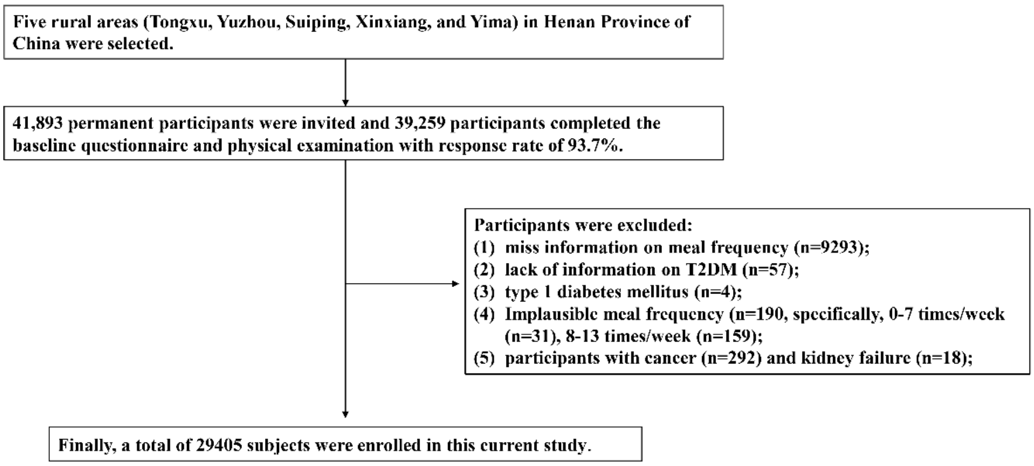

Figure S1. The flow chart for selecting study population
